# Lack of Spinal Neuropeptide Y Is Involved in Mechanical Itch in Aged Mice

**DOI:** 10.3389/fnagi.2021.654761

**Published:** 2021-05-28

**Authors:** Huan Cui, Wenliang Su, Yan Cao, Lulu Ma, Guangyan Xu, Wanying Mou, Hanlin Zhang, Jiawen Yu, Chao Ma, Xiuhua Zhang, Yuguang Huang

**Affiliations:** ^1^Department of Human Anatomy, Histology and Embryology, Neuroscience Center, Institute of Basic Medical Sciences, School of Basic Medicine, Chinese Academy of Medical Sciences, Peking Union Medical College, Beijing, China; ^2^Department of Anesthesiology, Peking Union Medical College Hospital, Chinese Academy of Medical Sciences, Peking Union Medical College, Beijing, China; ^3^Chinese Institute for Brain Research, Beijing, China

**Keywords:** neuropeptide Y, mechanical itch, spinal dorsal horn, senile pruritus, NPY1R

## Abstract

Neuropeptide Y (NPY) signaling plays an essential role in gating the pruritic afferent information in the spinal cord. Recent studies revealed that the aging process down-regulated the expression of NPY in the central nervous system. We propose that the lack of spinal NPY may be involved in certain types of pruritus in the elderly population. This study was designed to investigate the role of NPY in aging-induced itch using the senile mouse model. The expression of NPY in the spinal dorsal horn was compared between young (2 months old) and aged (24 months old) mice. Western blotting and immunohistochemistry showed that the expression of NPY was significantly reduced in the spinal dorsal horn in aged mice. In addition, a neuronal maker of apoptosis, TUNEL, was detected in the NPY positive neurons only in the aged spinal cord. Behavioral assay indicated that light mechanical stimulus evoked significantly more scratching in the aged than in the young mice, whereas chemical-evoked itch and pain-related behaviors were not altered. Intrathecal injection of either NPY or LP-NPY, a NPY receptor 1 (NPY1R) agonist, significantly alleviated the mechanically evoked itch in aged mice without altering the responses to chemical pruritogens. Our study suggested that downregulation of spinal NPY in the aged mice might play a role in the higher incidence of the mechanically evoked itch than that in the young mice. Therapies targeting the NPY system might serve as a potential strategy for alleviating the pruritic symptoms among the elderly population.

## Introduction

Pruritus is an increasingly concerning clinical problem, which frequently occurs in older people and causes needless suffering of patients’ lives. Senile pruritus is defined as chronic pruritus, which lasts 6 or more weeks in a person over 65 years old with elusive causes determined by appropriate examination (Ward and Bernhard, 2005; Valdes-Rodriguez et al., 2015). Previous studies showed a wide prevalence of senile pruritus, almost one-third in a nursing home in America. Increasing along with age, senile pruritus might be a part of the aging process (Chang et al., 2013; Clerc and Misery, 2017).

There are mainly two forms of itch: chemical itch irritated by chemical materials, such as histamine and chloroquine, and mechanical itch evoked by light mechanical stimuli (Fukuoka et al., 2013; Bautista et al., 2014; Koch et al., 2018). In addition, another type of itch can be evoked by audiovisual itch stimuli, which is assigned to contagious itch (Yu et al., 2017). The pathologic pruritus induced by innocuous mechanical stimuli is defined as “alloknesis” (Akiyama et al., 2012). Senile pruritus is usually associated with the dry skin condition and is always evoked by light mechanical stimulation, therefore presenting more likely as alloknesis (Berger et al., 2013; Clerc and Misery, 2017). The mechanism of senile pruritus remains unknown. Skin aging, charactered by the drier skin and weaker barrier, was mainly concerned in previous studies. Besides, the age-related loss of mechanosensitive Piezo2 channel in Merkel cells also produced alloknesis, suggesting that alteration in sensory element was involved in the senile pruritus (Feng et al., 2018).

Neuropeptide Y (NPY), a linear polypeptide with 36 amino acid residues, is one of the most abundant peptides in the central nervous system (CNS) (Tatemoto et al., 1982). The NPY system’s dysfunction is related to the cellular hallmarks of aging, including loss of proteostasis, deregulated nutrient sensing, mitochondrial dysfunction, cellular senescence, stem cell exhaustion, and altered intercellular communication (Tatemoto et al., 1982; Silva et al., 2005; Hsieh et al., 2013). Recent studies also indicated the specific role of spinal NPY in gating mechanical itch signal, but not chemical itch signal. The NPY^+^ interneurons formed an inhibitory pathway from hairy skin to suppress mechanical itch (Bourane et al., 2015). Furthermore, spinal NPY^+^ interneurons gated mechanical itch by inhibiting Urocortin 3^+^ (Ucn3^+^) excitatory interneurons that received peripheral inputs from Toll-like receptor 5^+^ (TLR5^+^) Aβ low-threshold mechanoreceptors (LTMRs) (Pan et al., 2019). In addition, NPY receptor 1 (NPY1R) signaling within the dorsal horn suppressed the activity of excitatory interneurons and gated mechanical itch after challenged by NPY (Acton et al., 2019).

However, the involvement of spinal NPY in senile pruritus is still unclear. Here, we sought to address the role of spinal NPY in aging-related alloknesis and alleviate senile pruritus through modulating the spinal NPY system. We hypothesized that aging induced the deficiency of NPY in the spinal dorsal horn, thereby evoking alloknesis in aged mice. We tested our hypothesis by observing alloknesis-related behavior in aged mice together with pharmacological intervention to NPY and NPY1R. Our findings revealed a neural mechanism for alloknesis in aged mice, suggesting potential therapeutic strategies for senile pruritus.

## Materials and Methods

### Animals

C57BL/6 mice (2 months old and 2 years old, 20–30 g, provided by HFK Bioscience Co., Ltd, Beijing, China) were housed in a controlled environment (21 ± 4°C, standard 12-h light/dark cycle, 4–5 mice per cage). All animal experiments were approved by the Institutional Animal Care and Use Committee in the Chinese Academy of Medical Sciences, Institute of Basic Medical Sciences (approval number: #211-2014). Both male and female mice were used in all studies. Animals were randomized to experimental groups, and no sex differences were noted. The gender of used mice in each group was included in the figure legends.

### Drug Administration

To observe the effect of neutralizing spinal NPY, rabbit isotype control IgG (5 μg in 5 μl sterile saline; Cell Signaling Technology) and rabbit anti-NPY IgG (5 μg in 5 μl sterile saline; Cell Signaling Technology) were intrathecally administrated 1 h prior to each behavioral test. NPY (Tocris) and [Leu31, Pro34]-NPY (LP-NPY, a selective agonist for NPY1R; Tocris) were dissolved in 0.9% sterile saline (vehicle) (Fuhlendorff et al., 1990). Both NPY (0.5 μg in 5 μl sterile saline) and LP-NPY (10 μg in 5 μl sterile saline) were intrathecally injected 15 min before each behavioral test (Hua et al., 1991; Acton et al., 2019). Both histamine and chloroquine were dissolved in sterile saline. Each drug injected was previously prepared and then coded by a laboratory assistant and not the experimenter. One experimenter who operated the drug injection was blinded to the code, and thus the chemical injected, as was another observer who carried out the behavioral tests.

### Mechanical Alloknesis Test

Our behavioral test followed a previous study (Feng et al., 2018). Briefly, mice were fully acclimated by being placed in the test chamber 1 h *quaque die* (QD) for 3 consecutive days before each behavioral assay. The fur on the nape of the neck was shaved without any skin lesions. Von Frey filaments ranging from 0.008 to 1.0 g were used to deliver mechanical stimuli. The filaments touched the skin and held on for up to 1 s unless the mice scratched the shaved skin with their hindpaw. Five stimuli were delivered for each filament with a 10-s interval between adjacent two weights. The total response number to each filament was counted to evaluate the degree of mechanical itch.

### Von Frey Test

According to previous reports, mice were placed on the wire mesh for 3 consecutive days to adapt to the environment before the test. The plantar surfaces of the hindpaw were stimulated with defined von Frey filaments (0.16 and 0.4 g) for five times with an interval of 10 min. The percentage of withdrawal response was calculated.

### Acute Itch Behavior

The nape of the neck was shaved for the tested mouse. The mice acclimated to the same chamber for 3 consecutive days. Histamine (200 μg in 10 μl normal saline per mouse) or chloroquine (50 μg in 10 μl normal saline per mouse) was applied to the shaved skin by intracutaneous injection. Immediately after each injection, mice were put into the chamber and videotaped for 30 min. The videotapes were played back, and an observer blinded to the treatments and groups counted the number of scratching bouts directed toward the injection sites.

### Immunohistochemistry and Immunofluorescence Staining

Under deep anesthesia with isoflurane, mice were perfused through the left ventricle with sterile 0.1 M phosphate-buffered saline (PBS), followed by pre-cooled 4% paraformaldehyde. The cervical spinal cords (C1–C3) were isolated. After a dehydrated overnight in 30% sucrose, tissues were embedded in OCT (Tissue-Tek) and cut into 15-μm thickness sections. Then, sections were permeabilized with 0.3% Triton X-100 and treated with microwave heat-induced antigen retrieval (95°C, 15 min). Furthermore, 5% hydrogen peroxide was applied to block the endogenous peroxidase. Then, sections were incubated with primary antibody (rabbit anti-neuropeptide Y, 1:400, Cat: 11976; Cell Signaling Technology) at 4°C overnight. After rinsing with PBS, sections were incubated with the second antibody (horseradish peroxidase-labeled goat anti-rabbit IgG, PV-9001; ZSGB-Bio, Beijing, China) and colored with diaminobenzidine (DAB) solution. Images were captured using a laser confocal microscopic imaging system (FV1000 and Olympus FluoView software; Olympus, Japan). ImageJ software was used to analyze the expression of NPY based on the color intensity. Quantitative analysis was determined by analyzing three spinal cords from young and aged mice (three sections per spinal cord).

For the immunofluorescence staining, sections were permeabilized with 0.3% Triton X-100, blocked for 1 h at room temperature with 10% donkey serum, and then incubated overnight at 4°C with primary antibody (rabbit anti-neuropeptide Y, 1:400, Cat: 11976; Cell Signaling Technology). After washing three times with PBS, sections were incubated by secondary antibody (Alexa Fluor 488-conjugated donkey anti-rabbit, 1:400, Cat: A-21206; Invitrogen, United States) for 1 h. From originally described in 1992, TUNEL staining has been used for localizing apoptotic DNA fragmentation *in situ* and detecting cell death *in vivo* (Gavrieli et al., 1992). Sections were washed in a darkroom and then incubated with the mixture of terminal deoxynucleotidyl transferase and Cy3-conjugated dUTP (One Step TUNEL Apoptosis Assay Kit; Beyotime, China) for 1 h. The slides were then washed in PBS and coverslipped with VECTASHIELD Mounting Medium with 4’,6-diamidino-2-phenylindole (DAPI, ZSGB-Bio, Beijing, China). The images were captured using a laser confocal microscopic imaging system (FV1000 and Olympus FluoView software; Olympus, Japan). Three non-adjacent spinal cord sections were randomly selected for each mouse, and three mice were included for each group. The TUNEL^+^ cells within the right spinal dorsal horn were counted for each section to evaluate the apoptotic condition. In addition, the percentages of TUNEL^+^ NPY^+^ cells among NPY^+^ neurons within the right spinal dorsal horn were summarized to evaluate the apoptotic condition of NPY^+^ neurons.

### Western Blot

The cervical spinal cords (C1–C3) in each group were excised. The samples were homogenized in T-PER Tissue Protein Extraction Reagent (Thermo Fisher Scientific, United States) containing Protein Phosphatase Inhibitor (Solarbio, Beijing, China) and phosphatase inhibitor Cocktail (CWBio, Beijing, China). The lysates were then homogenized and centrifuged (12,000 × *g* for 15 min at 4°C). The protein samples were then separated by sodium dodecyl sulphate (SDS)-polyacrylamide gel electrophoresis and transferred to a polyvinylidene fluoride (PVDF) membrane (Thermo Fisher Scientific, United States). The membranes were blocked in 5% (w/v) BSA for 1 h at room temperature and incubated with primary antibodies (rabbit anti-neuropeptide Y, 1:1,000, Cat: 11976; Cell Signaling Technology; mouse anti-β-actin, 1:1,000, Cat: CW0096; CWBio) overnight at 4°C, followed by incubation with corresponding secondary antibody for 1 h at room temperature. The bands were scanned with Tanon 5800 Luminescent Imaging Workstation (Tanon Science & Technology Co., Ltd. Shanghai, China) by High-sig ECL Western Blotting Substrate (Solarbio, Beijing, China). The band intensity was measured by ImageJ software (National Institutes of Health, Bethesda, MD, United States).

### Data Analysis

All data were presented as the mean and its standard error (mean ± SEM). Statistical analyses were performed using the SPSS software (version 17.0). Shapiro–Wilk test was applied to determine the normality for the parametric test. Differences between the two groups were analyzed using Student’s *t*-test. A statistically significant difference was defined as a two-sided *P* value < 0.05.

## Results

### Aging Downregulates the Expression of NPY in the Spinal Cord

**FIGURE 1:**
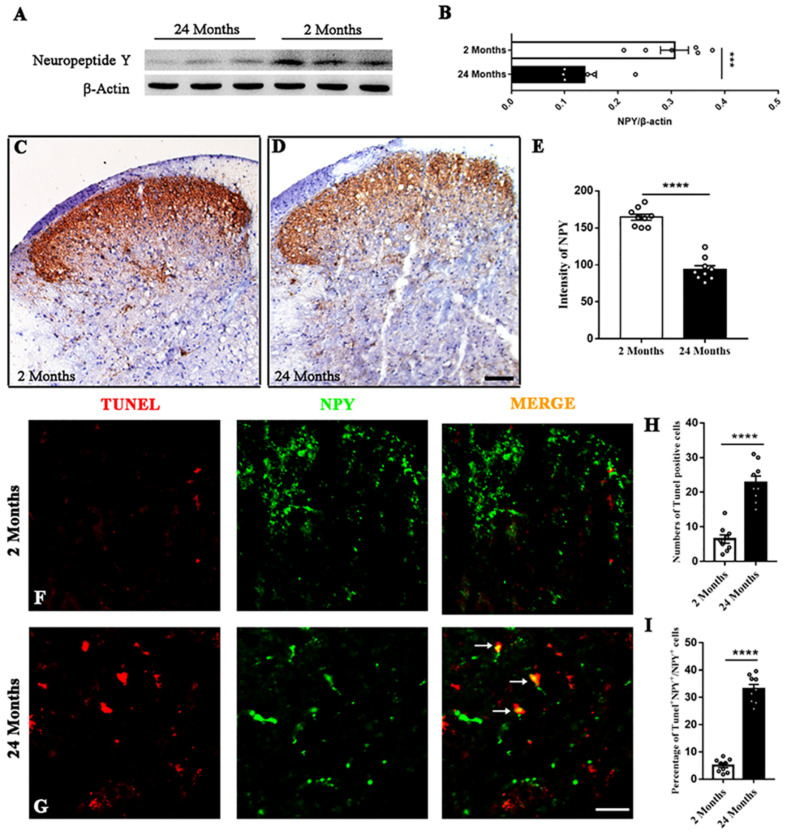
The degeneration of spinal NPY in aged mice. **(A,B)** Aging down-regulated NPY in the spinal dorsal horn. Six mice in each group, ****P < 0.0001, 2 (3 male and 3 female) versus 24 months (3 male and 3 female), Student’s *t*-test. **(C,D)** The distribution of NPY in the spinal dorsal horn of young **(C)** and aged **(D)** mice. Scale bar: 100 μm. **(E)** Quantitative analysis of NPY intensity indicated the degenerated spinal NPY in aged mice compared with the young mice. Nine slices from 3 mice in each group, ***P < 0.001, 2 (2 male and 1 female) versus 24 months (2 male and 1 female), Student’s *t*-test. **(F,G)** Colocalized cells (yellow, arrows) by TUNEL (red) and NPY (green) fluorescence in young and aged mice. Scale bar: 50 μm. **(H)** The number of TUNEL^+^ cells in each dorsal horn of young and aged mice. Nine slices from 3 mice in each group, ****P < 0.0001, 2 (2 male and 1 female) versus 24 months (2 male and 1 female), Student’s *t*-test. **(I)** The percentages of TUNEL^+^ cells among NPY^+^ cells in the spinal dorsal horn of young and aged mice. Nine slices from 3 mice in each group, ****P < 0.0001, 2 (2 male and 1 female) versus 24 months (2 male and 1 female), Student’s *t*-test.

The expression of spinal NPY was compared between young and aged mice. Western blotting revealed that the aging process significantly down-regulated NPY in the spinal dorsal horn (*P* = 0.0006, Figures 1A,B). The immunohistochemistry results showed that NPY was distributed mainly in the dorsal horn’s surface layer and the myelocoele area. The content of NPY in the spinal dorsal horn was reduced in the aged mice compared with the young mice (*P* < 0.0001, Figures 1C–E). The TUNEL staining in the aged mice showed more TUNEL^+^ cells than that in the young mice (*P* < 0.0001, Figures 1F–H). In addition, the immunofluorescence staining showed that part of the NPY^+^ cells was positive for TUNEL staining. Moreover, the percentage of TUNEL^+^ cell among the NPY^+^ cells was increased in the aged mice compared with the young mice (*P* < 0.0001, Figure 1I).

### Aging Induces Alloknesis Without Affecting the Chemical Itch

**FIGURE 2:**
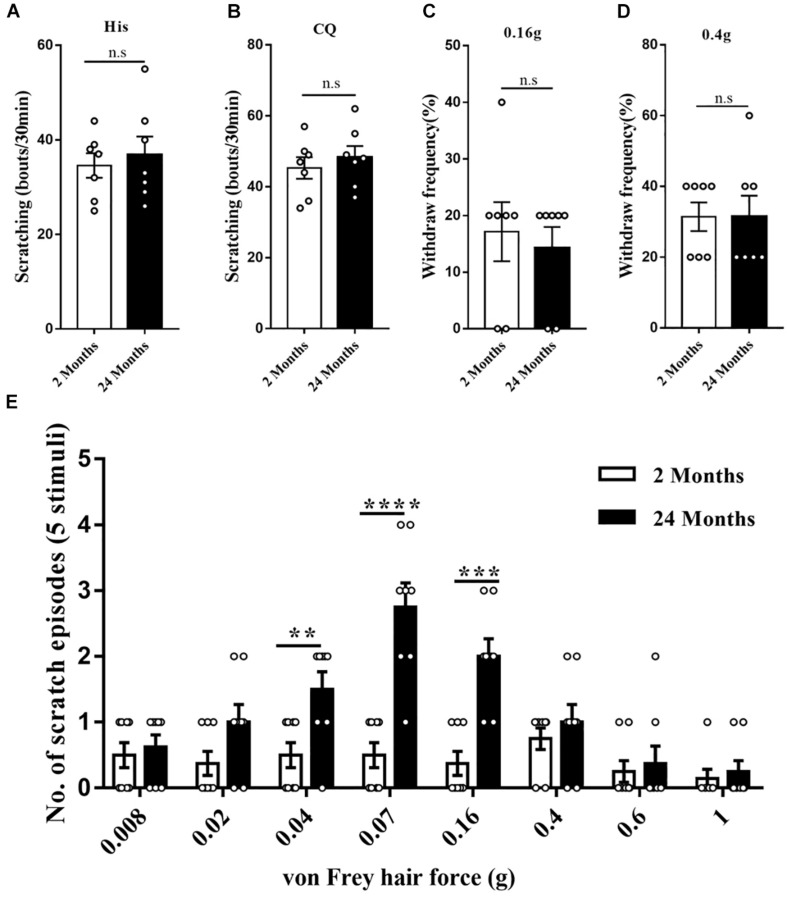
The enhanced mechanical itch in aged mice. **(A)** Scratching responses to intradermal injections of histamine in young (2 months, *n* = 7, 4 male and 3 female) and aged (24 months, *n* = 7, 4 male and 3 female) mice. n.s, not significant, Student’s *t*-test. **(B)** Scratching responses to intradermal injections of chloroquine in young (2 months, *n* = 7, 4 male and 3 female) and aged (24 months, *n* = 7, 4 male and 3 female) mice. n.s, not significant, Student’s *t*-test. **(C,D)** Percent paw withdrawal responses of young and aged mice to 0.16 **(C)** and 0.4 g **(D)** von Frey filament stimulation. *n* = 7 mice (4 male and 3 female) for each group. n.s, not significant, Student’s *t*-test. **(E)** Alloknesis scores in young (2 months, *n* = 8, 4 male and 4 female) and aged (24 months, *n* = 8, 4 male and 4 female) mice. **P < 0.01, ***P < 0.001, ****P < 0.0001, 2 versus 24 months, Student’s *t*-test.

Firstly, acute itch in response to classical pruritogens was assessed in young and aged mice. The scratching responses to histamine injection were not altered in the aged mice, suggesting that aging did not affect the histamine-dependent itch (*P* = 0.63, Figure 2A). Moreover, scratching response to intradermal injection of chloroquine was unaffected by aging, indicating the normal histamine-independent itch in aged mice (*P* = 0.51, Figure 2B). The response to mechanical pain stimulation toward the plantar surfaces of the hindpaw was also observed. Von Frey test (0.16 and 0.4 g) did not reveal any difference in the aged mice relative to the young mice (0.16 g: *P* = 0.66, 0.4 g: *P* > 0.99, Figures 2C,D). However, the mechanical stimulation to the nape skin by von Frey filaments, specifically the 0.04 (*P* = 0.0086), 0.07 (*P* < 0.0001), and 0.16 g (*P* = 0.00019) filaments, induced more scratching behavior in the aged mice (Figure 2E). These results indicated that aging sensitized mechanical itch in response to the light mechanical stimulation without affecting the acute chemical itch and mechanical pain.

### Neutralizing Spinal NPY Intensifies Alloknesis in Young and Aged Mice

**FIGURE 3:**
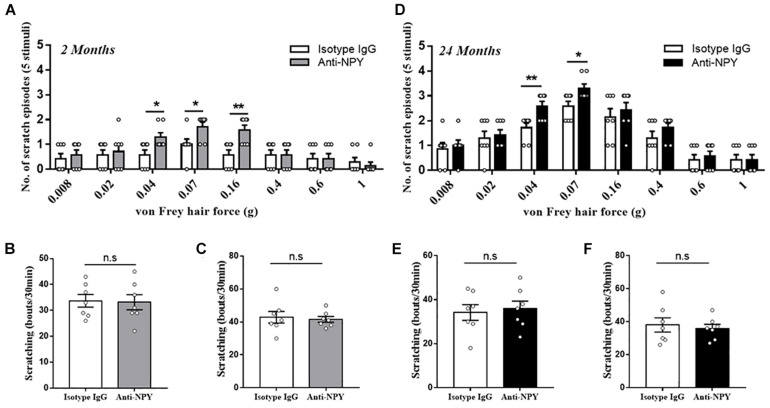
Neutralizing NPY induced mechanical itch. **(A)** Alloknesis scores in young mice receiving an intrathecal injection of isotype IgG (*n* = 7, 4 male and 3 female) and anti-NPY IgG (*n* = 7, 4 male and 3 female) mice. *P < 0.05, **P < 0.01, isotype IgG versus anti-NPY IgG, Student’s *t*-test. **(B)** Scratching responses to intradermal injections of histamine in young mice receiving an intrathecal injection of isotype IgG (*n* = 7, 4 male and 3 female) and anti-NPY IgG (*n* = 7, 4 male and 3 female) mice. n.s, not significant, Student’s *t*-test. **(C)** Scratching responses to intradermal injections of chloroquine in young mice receiving an intrathecal injection of isotype IgG (*n* = 7, 4 male and 3 female) and anti-NPY IgG (*n* = 7, 4 male and 3 female) mice. n.s, not significant, Student’s *t*-test. **(D)** Alloknesis scores in aged mice receiving an intrathecal injection of isotype IgG (*n* = 7, 4 male and 3 female) and anti-NPY IgG (*n* = 7, 4 male and 3 female) mice. *P < 0.05, **P < 0.01, isotype IgG versus anti-NPY IgG, Student’s *t*-test. **(E)** Scratching responses to intradermal injections of histamine in aged mice receiving an intrathecal injection of isotype IgG (*n* = 7, 4 male and 3 female) and anti-NPY IgG (*n* = 7, 4 male and 3 female) mice. n.s, not significant, Student’s *t*-test. **(F)** Scratching responses to intradermal injections of chloroquine in aged mice receiving an intrathecal injection of isotype IgG (*n* = 7, 4 male and 3 female) and anti-NPY IgG (*n* = 7, 4 male and 3 female) mice. n.s, not significant, Student’s *t*-test.

To investigate the effect of NPY deficiency, the neutralizing antibody was applied, and the relative behavioral outcomes were observed. For the young mice, the intrathecal injection of anti-NPY antibody induced alloknesis when stimulated by light mechanical stimulus (0.04 g: *P* = 0.023, 0.07 g: *P* = 0.028, and 0.16 g: *P* = 0.0044) compared with the isotype IgG (Figure 3A). However, the scratching response to chemical stimulus (histamine: *P* = 0.88 and chloroquine: *P* = 0.75) was not altered by the injection of anti-NPY antibody (Figures 3B,C). In addition, the behavioral effect of the anti-NPY antibody was evaluated in aged mice. Compared with the isotype IgG, the anti-NPY antibody increased the scratching behavior in aged mice receiving light mechanical stimulus (0.04 g: *P* = 0.0086 and 0.07 g: *P* = 0.023) (Figure 3D). Meanwhile, the scratching behavior in response to chemical agents (histamine: *P* = 0.74 and chloroquine: *P* = 0.68) showed no difference for the aged mice receiving isotype IgG and anti-NPY antibody (Figures 3E–F).

### Activating Spinal NPY1R Alleviates Alloknesis in Aged Mice

**FIGURE 4:**
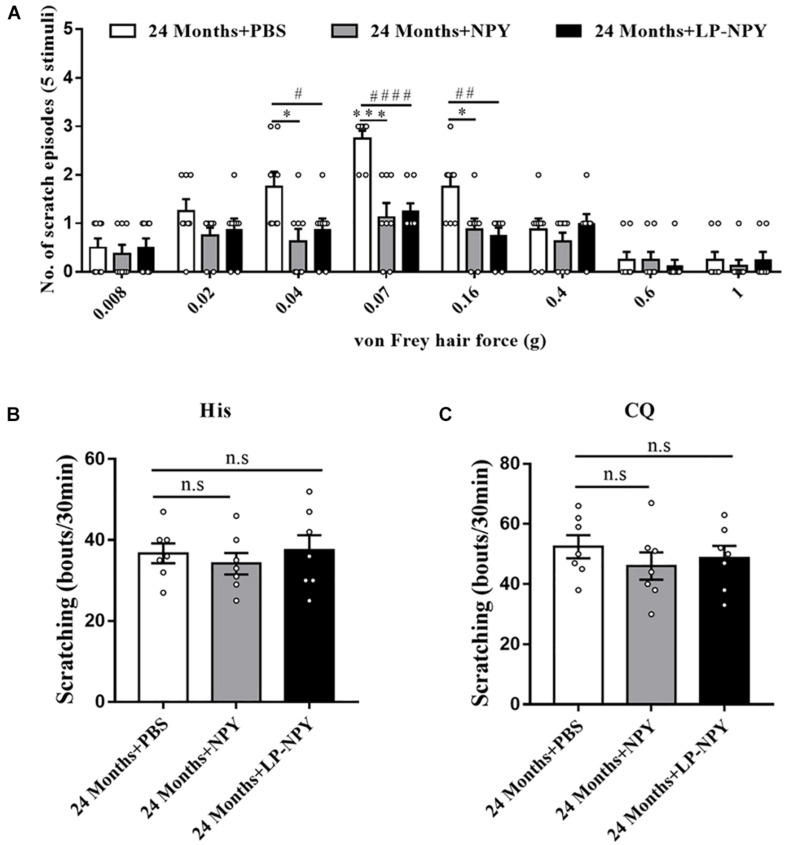
Activation of spinal NPY1R alleviated the mechanical itch in aged mice. **(A)** Alloknesis scores in 24 months + PBS (*n* = 8), 24 months + NPY (*n* = 8, 4 male and 4 female), and 24 months + LP-NPY (*n* = 8, 4 male and 4 female) mice. ^∗^*P* < 0.05, ^∗∗∗^*P* < 0.001, 24 months + PBS versus 24 months + NPY, Student’s t test; ^#^*P* < 0.05, ^##^*P* < 0.01, ^####^*P* < 0.0001, 24 months + PBS versus 24 months + LP-NPY, Student’s *t*-test. **(B)** Scratching responses to intradermal injections of histamine in 24 months + PBS (*n* = 7, 4 male and 3 female), 24 months + NPY (*n* = 7, 4 male and 3 female), and 24 months + LP-NPY (*n* = 7, 4 male and 3 female) mice. n.s, not significant, Student’s *t*-test. **(C)** Scratching responses to intradermal injections of chloroquine in 24 months + PBS (*n* = 7, 4 male and 3 female), 24 months + NPY (*n* = 7, 4 male and 3 female), and 24 months + LP-NPY (*n* = 7, 4 male and 3 female) mice. n.s, not significant, Student’s *t*-test.

The NPY system’s role in regulating mechanical itch was further assessed by intrathecally administering NPY or the selective NPY1R agonist LP-NPY to the aged mice. The behavior assay showed that a supplement of NPY alleviated the mechanical itch in aged mice, especially in the 0.04 (*P* = 0.016), 0.07 (*P* = 0.00027), and 0.16 g (*P* = 0.021) von Frey filaments. Moreover, activating NPY1R by LP-NPY reduced the scratching response to light mechanical stimuli (0.04 g: *P* = 0.040, 0.07 g: *P* = 0.000014, and 0.16 g: *P* = 0.0048) (Figure 4A). However, neither the injection of NPY nor LP-NPY altered the reaction to classical pruritogens, namely, histamine and chloroquine, suggesting the unaffected chemical itch (His: 24 months + PBS vs. 24 months + NPY, *P* = 0.49, 24 months + PBS vs. 24 months + LP-NPY, *P* = 0.88; CQ: 24 months + PBS vs. 24 months + NPY, *P* = 0.30, 24 months + PBS vs. 24 months + LP-NPY, *P* = 0.51, Figures 4B,C).

## Discussion

In this study, we first demonstrated that aging down-regulated NPY in the spinal dorsal horn, that NPY deficiency evoked mechanical itch in young mice and intensified alloknesis in aged mice, and that pharmacological activation of the NPY/NPY1R signaling alleviated alloknesis in aged mice.

NPY was first abstracted from the pig brain in 1982 (Tatemoto et al., 1982). Accumulating studies had suggested that the NPY system, especially in the brain, was linked to the aging process and lifespan determination (Hua et al., 1991; Peng et al., 1993; Michalkiewicz et al., 2003; Gehrig et al., 2012). In general, NPY was down-regulated by aging in multiple brain regions, such as the cortex, hypothalamus, and striatum. In this study, the protein level of NPY was significantly decreased in the spinal dorsal horn of aged mice. Moreover, these NPY-positive neurons presented the apoptotic character. Collectively, the deficiency of NPY might be the special label of aging in the CNS.

Actually, NPY defines a unique population of spinal inhibitory interneurons, most of which displayed a tonic firing pattern following current injection and expressed the markers of inhibitory interneuron. Moreover, NPY^+^ interneurons gated mechanical itch specifically without affecting chemical itch (Bourane et al., 2015). In the present study, aged mice presented mechanical alloknesis together with degenerated NPY^+^ neurons in the spinal dorsal horn, while the acute itch induced by classical pruritogens was not affected. This result suggested the correlation between NPY deficiency and alloknesis in aged mice. In aged mice, intrathecal injection of NPY alleviated mechanical itch suggesting that leaky gate control for mechanical itch within the spinal dorsal horn could be repaired by the supplement of NPY, while neutralizing spinal NPY by antibody intensified alloknesis. Previous studies also revealed the excitatory circuit for mechanical itch: Ucn3^+^ interneurons received inputs from TLR5^+^ Aβ LTMRs and were directly gated by spinal NPY^+^ interneurons (Pan et al., 2019). The changes within the excitatory circuit induced by aging and relative effects on aging alloknesis should be further investigated in the future. Considering that multiple neuropeptides serve as the gate for nociceptive information in the spinal cord (Kardon et al., 2014; Sun et al., 2017) and neuropeptides intend to degenerate during the aging process, the role of neuropeptides in somatosensory dysfunction among the aged population could be further investigated in the future.

The NPY receptors belong to G protein-coupled receptors (GPCR), including NPY1R, NPY2R, NPY4R, and NPY5R. Based on previous studies, this function of NPY might act by suppressing the excitability of excitatory intermediate neurons by the inhibitory signal from NPY1R (Acton et al., 2019). To determine the effective receptor for the NPY injection, the selective agonist for NPY1R was used to activate NPY1R specifically. The alleviation of alloknesis after the intrathecal application of LP-NPY in aged mice suggested, at least partly, that the responsive receptor for NPY was NPY1R. However, the chemical itch induced by histamine and chloroquine was not affected in the aged mice receiving the injection of NPY and LP-NPY. NPY1R was known as the major alloknesis-related NPY receptor now, so pharmacological modulation only targeted NPY1R in the present study. However, the responsive receptor might be multiple, so the effects of other NPY receptors on aging alloknesis should be studied further.

The somatosensory dysfunction, especially itch and pain, among the aged population burdens public health (Berger et al., 2013; Blyth and Noguchi, 2017; Qiu et al., 2020). Our findings illuminated that spinal NPY deficiency induced the loss of mechanical itch gating in aged mice, which shed new light on therapeutic strategies for the treatment of senile pruritus.

## Data Availability Statement

The original contributions presented in the study are included in the article/supplementary material, further inquiries can be directed to the corresponding authors.

## Ethics Statement

The animal study was reviewed and approved by Institutional Animal Care and Use Committee in Chinese Academy of Medical Sciences and Institute of Basic Medical Sciences.

## Author Contributions

HC and WS drafted the manuscript and performed the behavioral assessment of pain and itch. HC performed the immunofluorescence staining and western blotting. YC, LM, GX, WM, HZ, and JY performed the data analysis and behavioral assay. CM, XZ, and YH conceived the study, participated in its design, and helped to draft the manuscript. All authors read and approved the final manuscript.

## Conflict of Interest

The authors declare that the research was conducted in the absence of any commercial or financial relationships that could be construed as a potential conflict of interest.

## Abbreviations

NPY: neuropeptide Y
NPY1R: neuropeptide Y receptor 1
TLR5: Toll-like receptor 5
Ucn3: Urocortin 3.
